# Clinical and molecular features of patients with amyotrophic lateral sclerosis and *SOD1* mutations: a monocentric study

**DOI:** 10.3389/fneur.2023.1169689

**Published:** 2023-05-17

**Authors:** Delia Gagliardi, Paolo Ripellino, Megi Meneri, Roberto Del Bo, Sara Antognozzi, Giacomo Pietro Comi, Claudio Gobbi, Antonia Ratti, Nicola Ticozzi, Vincenzo Silani, Dario Ronchi, Stefania Corti

**Affiliations:** ^1^Neuroscience Section, Department of Pathophysiology and Transplantation (DEPT), Dino Ferrari Centre, University of Milan, Milan, Italy; ^2^Neurology Unit, Foundation IRCCS Ca’ Granda Ospedale Maggiore Policlinico, Milan, Italy; ^3^Department of Neurology, Neurocenter of Southern Switzerland EOC, Lugano, Switzerland; ^4^Neuromuscular and Rare Diseases Unit, Foundation IRCCS Ca’ Granda Ospedale Maggiore Policlinico, Milan, Italy; ^5^Faculty of Biomedical Sciences, Università della Svizzera Italiana, Lugano, Switzerland; ^6^Department of Neurology and Laboratory of Neuroscience, IRCCS Istituto Auxologico Italiano, Milan, Italy; ^7^Department of Medical Biotechnology and Translational Medicine, Università degli Studi di Milano, Milan, Italy

**Keywords:** amyotrophic lateral sclerosis, superoxide dismutase, *SOD1*-ALS, cohort, *SOD1* variants

## Abstract

**Introduction:**

*SOD1* was the first gene associated with both familial and sporadic forms of amyotrophic lateral sclerosis (ALS) and is the second most mutated gene in Caucasian ALS patients. Given their high clinical and molecular heterogeneity, a detailed characterization of *SOD1*-ALS patients could improve knowledge about the natural history of this disease. Here, the authors aimed to provide a clinical and molecular description of a monocentric cohort of *SOD1*-ALS patients.

**Methods:**

Amyotrophic lateral sclerosis (ALS) patients referring to the neurology unit of our center between 2008 and 2021 were clinically assessed and underwent molecular testing for *SOD1*. Segregation studies in available family members and *in silico* analysis were performed to sustain the pathogenicity of the identified *SOD1* variants.

**Results:**

Among the 576 patients in our cohort, we identified 19 individuals harboring a mutation in *SOD1* (3.3%), including 15 (78.9%) with a familial and four (21.1%) with a sporadic form. The spinal onset of the disease was observed in all patients, and survival was extremely variable, ranging from 8 months to over 30 years. Twelve different *SOD1* missense variants were identified in our cohort, including one novel mutation (p.Pro67Leu).

**Discussion:**

In the present series, we provided the first description of an Italian monocentric cohort of *SOD1*-ALS patients, and we expanded the repertoire of *SOD1* mutations. Our cohort presents several remarkable features, including variable expressivity in the same family, atypical presentation (ataxia, cognitive impairment, and other extra-motor symptoms), and different modes of inheritance of a given mutation in the same family. Given the recent authorization of *SOD1*-directed antisense oligonucleotide for use in *SOD1*-ALS patients, we recommend prompt screening for *SOD1* mutations in novel ALS patients with familiar or sporadic presentations.

## 1. Introduction

*SOD1,* encoding the copper–zinc superoxide dismutase, was the first gene to be associated with amyotrophic lateral sclerosis (ALS) (1), and it is currently the second most common genetic cause of ALS, after the *C9ORF72* expansion, in Caucasian patients (2). Thus far, more than 200 pathogenic mutations in *SOD1* have been identified in patients with ALS^1^,^2^,^3^ (3) and, overall, they account for up to 20% of familial forms (fALS) and 1%–2% of sporadic cases (sALS) (4). Most of them are heterozygous dominantly inherited variants, expected to exert their pathogenetic effect through a toxic gain of function mechanism. Indeed, several *in vitro* and *in vivo* studies have shown that the pathogenicity of *SOD1* mutations is due to misfolded cytoplasmic accumulation, resulting in intracellular aggregates (5–7). Conversely, the loss of function of the SOD1 protein is not associated with a neuromuscular phenotype in the transgenic *SOD1*-mouse model (8), and its contribution to disease pathogenesis is still debated (9).

Seven *SOD1* mutations (p.Leu85Phe, p.N87Ser, p.Asp91Ala, p.Leu118Val, p.Leu127Ser, p.Leu145Ser, and p.Gly28delGGACCA) have been described in a homozygous state, but only a few of them produce a recessive pattern of inheritance (9, 10). The p.Asp91Ala variant, the most common *SOD1* mutation worldwide, is inherited as a recessive trait in Scandinavians, while it displays a dominant pattern in other populations, although its pathogenic role in heterozygosis is still debated (10).

Clinical phenotype in *SOD1*-related ALS (*SOD1*-ALS) is heterogenous, but it is frequently associated with the spinal onset and lower limb involvement, rare cognitive impairment, and slow progression. Familial and sporadic forms are clinically indistinguishable.

Describing the natural history of ALS patients harboring a mutation in one ALS causative gene is particularly relevant given the advent of new therapeutic approaches based on gene modulation, such as antisense oligonucleotides (ASOs) (11–13). The intrathecal administration of the *SOD1*-targeting ASO Tofersen has yielded promising results in phase-3 clinical studies, which have led to the authorization of an ongoing early access program for the use of Tofersen in all individuals with *SOD1*-ALS (14, 15).

Here, we describe the findings relative to our monocentric cohort of patients with genetically confirmed *SOD1*-ALS, focusing on the most atypical forms. Among these, we report a novel variant (p.Pro67Leu), causing an extremely slow-progressing lower motor neuron (LMN) involvement, and the coexistence of ALS and atypical presentation (i.e., ataxia and cognitive impairment) in the same family.

## 2. Materials and methods

### 2.1. Data collection

We enrolled patients referred to the Neurology Unit of Fondazione IRCCS Ca′ Granda Ospedale Maggiore Policlinico of Milan between 2008 and 2021 with a diagnosis of possible, probable, or definite ALS according to Awaji-Shima criteria (16) and who tested positive for a mutation in *SOD1*. Gender, age and site of onset, family history of ALS, disease duration until death, presence of bulbar and respiratory involvement, and presence of extra-motor features were collected.

### 2.2. Genetic analysis

Genomic DNA was extracted from peripheral blood samples according to standard procedures (Flexi Gene DNA Handbook, Qiagen). Coding regions of *SOD1* (NM_000454.4) were analyzed by PCR analysis followed by direct sequencing on an ABI Prism 3130 instrument in the patients included in this study (primers are listed in Supplementary Table 1). *SOD1* mutations were also analyzed in available family members by direct sequencing.

### 2.3. *In silico* analysis and clinical prediction

To predict the deleterious effects of all *SOD1* variants identified in our cohort, we employed the meta-predictor tool REVEL (17). The classification of the identified variants (“pathogenic,” “likely pathogenic,” “uncertain significance,” “likely benign,” and “benign”) was performed according to the American College of Medical Genetics and Genomics (ACMG) criteria (18).

## 3. Results

### 3.1. Description of the *SOD1*-ALS cohort

Five hundred and seventy-six patients with ALS, of whom 69 fALS (12%), were referred to our center during the inclusion period and were all tested for *SOD1* mutations. We identified 19 *SOD1*-ALS cases (9 male patients and 10 female patients) from 15 independent families (3.3%), including 15 fALS (21.7% of all familial forms and 78.9% of *SOD1*-ALS) and four sALS cases (0.8% of all sporadic forms and 21.1% of *SOD1*-ALS). All patients with *SOD1*-ALS were negative for pathogenic expansion in the *C9ORF72* gene and for variants in *TARDBP* and *FUS* mutational hotspots (exon 6 of *TARDBP* and exons 13-14-15 of *FUS*).

Demographic and clinical features of *SOD1*-ALS patients are listed in Table 1. The median age at onset was 50 years (ranging from 18 to 74 years). Spinal onset was observed in all patients: a total of 14 individuals started with lower limb disturbances, while five presented with upper limb symptoms. Six patients developed bulbar symptoms during the disease course. Respiratory involvement was detected in six patients. Disease duration is not available for all patients but is extremely variable, ranging from 8 months to more than 30 years.

**Table 1:** Clinical and molecular description of the *SOD1*-ALS cohort.

Patient	Variant	Base change	Zygosity	First description	fALS	Sex	Native country	Age at onset (ys)	Site of onset	Disease duration (ys)	Bulbar/respira tory	Extramotor features
1.1	p.Gly13Arg	c.37G > C	Het	Penco et al. (19)	+	M	Macedonia	30	LL	>10–alive	+/+	−
1.2	p.Gly13Arg	c.37G > C	Het	Penco et al. (19)	+	M	Macedonia	55	LL	>6–alive	+/−	Gait ataxia, extrapiramidal symptoms (rigidity and bradykinesia), cognitive impairment, apraxia, and depression
2	p.Glu22Gly	c.65A > G	Het	Syriani et al. (20)	−	F	Switzerland	38	UL	>30	+/+	−
3	p.Gln23Arg	c.68A > G	Het	Corti et al. (21)	+	M	Bangladesh	38	LL	>6	+/−	−
4	p.Pro67Leu	c.200C > T	Het	Present study	−	F	Italy	35	LL	>21–alive	−/−	Subjective balance impairment
5	p.Pro67Ser	c.199C > T	Het	Keckarevic et al. (22)	+	M	Switzerland	74	UL	8 months	−/+	−
6	p.Asp91Ala	c.272A > C	Het	Robberecht et al. (23)	−	F	Italy	52	LL	>14–alive	−/−	Lower limb dysestesia
7	p.Asp91Ala	c.272A > C	Het	Robberecht et al. (23)	−	M	Italy	47	UL	>16–alive	+/+	−
8	p.Ala96Thr	c.286G > A	Het	Gellera et al. (24)	+	F	Italy	59	LL	>4–alive	−/−	Presymptomatic iperckemia
9	p.Leu107Val	c.319C > G	Het	Juneja et al. (25)	+	F	Macedonia	33	UL	n.a.	n.a.	n.a.
10	p.Leu118Val	c.352C > G	Hom	Synofkiz et al. (26)	+	M	Syria	44	LL	9	−/+	Muscle pain
11.1	p.Glu122Gly	c.365A > G	Het	Canosa et al. (27) and Dangoumau et al. (28)	+	M	Italy	50	LL	9	−/−	Sensory disturbance, sphincteric dysfunction
11.2	p.Glu122Gly	c.365A > G	Het	Canosa et al. (27) and Dangoumau et al. (28)	+	M	Italy	54	UL	> 5–alive	−/−	−
12	p.Leu145Phe	c.435G > C	Het	Masè et al. (29)	+	F	Italy	66	LL.	n.a.	n.a.	−
13	p.Leu145Phe	c.435G > C	Het	Masè et al. (29)	+	F	Italy	51	LL	n.a.	n.a.	−
14.1	p.Leu145Ser	c.434 T > C	Hom	Gagliardi et al. (30)	+	F	Iran	33	LL	2	+/+	Toe paresthesia, muscle pain
14.2^*^	p.Leu145Ser	c.434 T > C	Hom	Gagliardi et al. (30)	+	M	Iran	18	LL	9	+/+	−
14.3^#^	p.Leu145Ser	c.434 T > C	Het	Sapp et al. (31)	+	F	Iran	64	LL	>1–alive	−/−	Ataxia and lower limb numbness
15	p.Leu145Phe	c.435G > C	Het	Deng et al. (32)	+	F	Bulgaria	62	LL	n.a.	−/−	−

ALS, amyotrophic lateral sclerosis; fALS, familial ALS; Het, heterozygous; Hom, homozygous; LL, lower limb; UL, upper limb. Variants are classified according to the nomenclature NM_000454 for the cDNA and NP_000445.1 for the protein. *, brother of 14.1; #, mother of 14.1. ° , brother of 11.1; §, father of 1.1.

**Figure 1:**
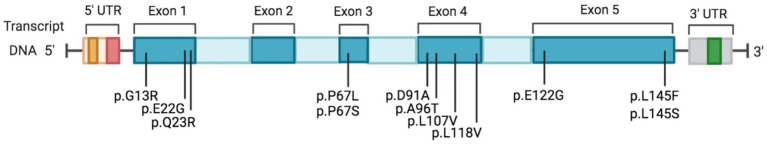
Scheme of *SOD1*: the mutations identified in our cohort of ALS patients are indicated.

Twelve *SOD1* missense variants were identified in our cohort (Table 1; Figure 1). All but one (p.Pro67Leu) have been previously reported in association with ALS. Most variants were dominantly inherited. One of them (p.Leu145Ser) occurred both in heterozygous and homozygous states in the same family, as previously described (30). The pathogenic allele p.Leu118Val has been described in homozygosis (26). The variant p.Glu22Gly, carried by a woman with a disease duration higher than 30 years, has been already associated with a long survival time (20).

American College of Medical Genetics and Genomics criteria classified the variants as pathogenic or likely pathogenic with the exceptions of p.Asp91Ala and p.Leu118Val which were classified as variants of uncertain significance (Supplementary Table 2).

Clinical and molecular findings of selected probands, presenting atypical features, are described below.

### 3.2. Patient 4 (p.Pro67Leu)

A 52-year-old woman presented with a 17-year history of a gradual gait disturbance starting from the right lower limb and increasingly progressing to the left lower limb. The symptoms were followed by loss of dexterity and fine hand movement impairment due to a progressive distal upper limb weakness. Muscle cramping and fasciculation in the four limbs were noted. By the age of 48 years, she complained about difficulty in climbing stairs.

**Figure 2:**
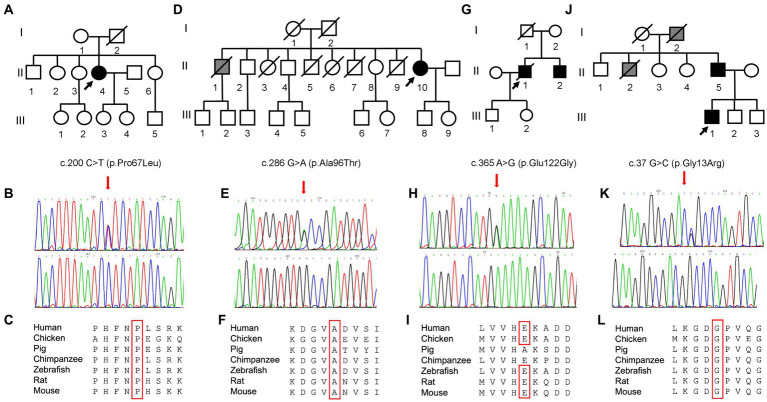
Pedigree and molecular findings of four selected ALS cases. **(A,D,G,J)** Pedigrees of the families carrying the missense mutations in the *SOD1* gene. Roman and Arab numbers are used to indicate the generation and the subject within each generation. The arrow indicates the proband, carrier of the missense mutation in the *SOD1* gene. Black symbols indicate disease status (diagnosis of ALS); gray symbol indicates the presence of a disabling neuromuscular disease without a confirmed clinical and genetic diagnosis of ALS. **(B,E,H,K)** Electropherograms showing *SOD1* variants (red arrows) c.200\u00B0C > T (p.Pro67Leu), c.286 G > A (p.Ala96Thr), c.365A > G (p.Glu122Gly), and c.37 G > C (p.Gly13Arg) as detected in patients (top) compared to control individuals (bottom). **(C,F,I,L)** Conservation of the amino acid positions affected by mutations across different species.

Family history was negative for neuromuscular disorders (Figure 2A). The proband is the fourth of five siblings, the patient’s father died at 43 years of age due to leukemia, and the patient’s mother is 88 years and in good health.

The patient’s neurological evaluation showed limb weakness with a distal to proximal gradient, diffuse muscle wasting, and hyperreflexia in the four limbs. Bulbar and respiratory functions were normal. Brain and spinal cord magnetic resonance imaging (MRI) were unremarkable. Electromyography (EMG) showed moderate neurogenic changes in the four limbs and the dorsal paraspinal muscles and acute denervation in the lumbar and cervical regions, consistent with a diagnosis of ALS. The patient was started with riluzole. Presently, at the age of 56 years, she walks with assistance and does not demonstrate bulbar or respiratory involvement.

Molecular analysis of the *SOD1* gene in this patient revealed the heterozygous substitution c.200C > T in the exon 3, resulting in the amino acid change p.Pro67Leu (Figures 2B,C). The first-degree relatives of the patients are currently in good health and refused to undergo the genetic testing.

To the best of our knowledge, this variant has never been described before, and it is not reported in genomic databases (gnomAD, 1000GP, ExAC).

Proline residue at codon 67 is highly conserved among various species. A different substitution in the same amino acid position (p.Pro67Ser) has been reported in seven ALS cases, of whom four were familial, with a heterogenous age at onset (21–72 years) and survival between 6 and 48 months (12, 22, 33–36). Similarly, p.Pro67Ala and p.Pro67Arg have been associated with a slow progression and a long disease duration (37–39). Recently, a compound heterozygous p.Pro67Ser/Asp91Ala was found in a 69-year-old ALS patient with a facial onset and slow progression (40). We also identified the p.Pro67Ser change in the patient (patient 5) presenting at 75 years of age with an 8-month history of progressive weakness in the four limbs and a positive family history of neuromuscular disorder in his grandfather and his sister, who died at the age of 50 and 68, respectively. Pulmonary evaluation detected a restrictive respiratory failure. The death occurred after 8 months from symptom onset due to a cardiac attack.

### 3.3. Patient 8 (p.Ala96Thr)

A 61-year-old woman, with a 10-year history of increased creatine kinase (CK) level (mean value 500 U/L), developed a gradual and progressive gait disturbance characterized by fatigability, paraparesis with difficulty in climbing stairs, and cramps. After 2 years, she noticed increasing distal weakness in the upper limbs. One of her older brothers was diagnosed with asymptomatic hyperCKemia and underwent a muscle biopsy, which was unremarkable. He died for unrelated causes prior to patient’s presentation to medical attention (Figure 2D). Five of her siblings died in childhood of unknown causes.

Neurological examination revealed reduced deep tendon reflexes (DTRs) and more distal than proximal weakness in the four limbs, without pyramidal signs. EMG showed chronic neurogenic changes with acute denervation in upper and lower limb muscles and paraspinal muscles. Motor-evoked potentials (MEPs) at the lower limb showed an absent cortical response. Brain and spinal cord MRI ruled out other diseases. Neurogenic alterations were found at muscle biopsy, compatible with a slow-progressing form of motor neuron disease (MND). The heterozygous change c.286G > A, resulting in the substitution of alanine with threonine in position 96, was found in *SOD1* gene (Figures 2E,F).

The p.Ala96Thr variant in *SOD1* has been previously found in two unrelated Italian ALS patients with a sporadic presentation (24, 41), but its role in causing the disease has been debated. The first mutation was found in a 26-year-old man with upper limb presentation and a slow-progressing disease (24). The same missense mutation was also detected in the unaffected mother and in two unaffected siblings, arguing against a pathogenic role in the proband. However, incomplete penetrance or the contribution of other genetic modifiers cannot be excluded, as shown in other *SOD1* mutations (42). The second patient is a 45-year-old woman with an extremely slow-progressing disease, which had started in lower limbs and is still not involving the bulbar district after 20 years (41).

Here, we described the third report of a p.Ala96Thr *SOD1* mutation in an Italian patient with a spinal onset and slow-progressing ALS. Although the genetic analysis on the proband’s parents cannot be performed, this finding increases the likelihood that p.Ala96Thr might be a disease-associated mutation and opens the path to further studies to characterize this variant.

### 3.4. Patients 11.1 and 11.2 (p.Glu122Gly)

A 49-year-old man came to medical attention for a slowly progressive gait disorder, characterized by lower limb stiffness, followed by a burning sensation. He was investigated for chronic inflammatory demyelinating polyneuropathy, but cerebrospinal fluid (CSF) analysis and muscle biopsy resulted normal. By the age of 52 years, he gradually developed tactile and thermal hypoesthesia below level D10; neurological evaluation showed lower limb mild proximal weakness, absent bilateral ankle and patellar DTRs, and bilateral Babinski reflexes. He underwent a spinal cord MRI, lumbar puncture, and spinal angiography to rule out a spinal cord pathology. EMG showed neurogenic alterations in lower limbs, while MEPs proved a pathological central motor conduction time. Over the following years, he developed urinary incontinence, upper limb weakness, and progressive gait disturbance until paraplegia.

Despite presenting extra-motor symptoms at onset (sensory and sphincteric symptoms), neurophysiological findings were consistent with a slowly progressive MND. Molecular analysis for *SOD1* identified a point mutation (c.365A > G) at codon 122, leading to the substitution of glutamic acid with glutamine. The proband’s father died at 40 years of gastric cancer, while his healthy mother tested negative. His 54-year-old brother with a recent onset of left-hand weakness and muscle wasting was found to carry the same mutation identified in the proband.

The p.Glu122Gly mutation was first described in two independent sporadic patients: an Italian man with a 14-year disease course and a prevalent lower motor neuron involvement at lower limbs (27) and a woman with slowly progressing gait disturbance, followed by bulbar impairment (28). Symptoms started at 70 years of age in both patients.

The localization of Glu122 residue next to histidine which is involved in the interaction with copper suggests a possible pathogenic role, supported by the findings from *in silico* analysis. Moreover, *SOD1*-E122G-expressing NSC34 and the primary culture of mouse MNs showed the presence of aggregates positive for SOD1 and ubiquitin and reduced cell viability under oxidative stress (28), proving a pathogenic link between ALS and this variant.

### 3.5. Patients 1.1 and 1.2 (p.Gly13Arg)

A Macedonian 30-year-old athletic man presented to medical attention for the occurrence of cramps and fasciculations in the lower limbs. He became unable to run and climb stairs within 1 year. Neurological examination showed diffuse fasciculations, mild distal weakness in lower limbs, increased DTRs, and bilateral Babinski signs. EMG showed spontaneous activity (fibrillations and fasciculations) in three regions (cervical, brachial, and lumbosacral) with bulbar sparing. MEPs showed increased central conduction time and dispersed responses with reduced amplitude. Brain MRI (T2-weighted sequences) showed mild hyperintensity of the bilateral corticospinal tract. The genetic test for spinal bulbar muscular atrophy (SBMA) was negative. The patient was therefore diagnosed with ALS.

The family tree (Figure 2G-J) suggested an autosomal-dominant neuromuscular disease. His grandfather and his uncle developed leg weakness by the age of 55 years, became wheelchair-bound by 60 years, and died at nearly 65 years of age of pneumonia and respiratory failure. The genetic analysis revealed a glycine–arginine substitution (p.Gly13Arg) in exon 1 of the *SOD1* gene (Figures 2K,L). The patient became wheelchair-dependent by 36 years and developed bulbar and respiratory symptoms (mild orthopnea, dysarthria) by the age of 40 years. In March 2022, he started treatment with Tofersen.

The proband’s father was hospitalized several years later for a history of unexplained and repeated falls, by the age of 55 years. Neurological examination showed occasional fasciculations in the calves (confirmed by EMG), with normal muscle strength and trophism and normal DTRs and flexor plantar responses. However, the patient was unable to walk without assistance due to a notable gait ataxia. The pull test was positive, and finger-to-nose and heel-to-knee tests were impaired. Mild bradykinesia and axial rigidity were present. His family members described behavioral changes occurring in the last years. Detailed neuropsychological evaluation evidenced severe, multidomain cognitive deficits, with ideomotor apraxia and reduced verbal fluency and comprehension despite relatively preserved short-term memory. CSF examination, including dosage of tau, phospho-tau, and Aβ-42, was normal, and serum and CSF anti-onconeural and anti-GAD antibodies were negative. Brain MRI displayed thinning of the sulci in the bilateral parietal cortex. Finally, the genetic test revealed the same mutation in the *SOD1* gene detected in his son.

This variant was previously reported in one Italian family and associated with an extremely slow rate of progression (19, 24) with onset at nearly 60 years and with the involvement of the distal muscles of the lower limbs, resulting in gait disturbance. The most peculiar finding in this family was the clinical presentation of ataxia and cognitive deficits without overt signs of MND in the proband’s father. The ataxic gait with repeated falls has been reported in a patient with fALS and a p.Glu100Lys variant in *SOD1* (43). Early cognitive dysfunction, together with bulbar involvement, has been recently described in two unrelated families harboring a p.N66Thr mutation in *SOD1* (44) and has been considered an exceptional finding in *SOD1*-ALS so far.

## 4. Discussion

In the present series, we provide the first description of an Italian monocentric cohort of *SOD1*-ALS patients, and we added a novel variant (p.Pro67Leu) to the repertoire of *SOD1* variants associated with ALS. Patient 4 presented with early-onset lower motor neuron syndrome, main lower limb involvement, and slow progression, similar to the clinical phenotype reported in p.Pro67Ala, p.Pro67Arg, and p.Pro67Ser carriers. Moreover, *in silico* analysis predicted this amino acid change to be deleterious, in accordance with the high conservation of proline in position 67 across species. These remarks suggest that p.Pro67Leu represents a novel pathogenic *SOD1* variant.

Moreover, we provided the third report of a p.Ala96Thr variant in an Italian carrier with signs and symptoms compatible with a slowly progressing MND. Despite the occurrence of the same variant in asymptomatic individuals in previous reports and the lack of evidence of a proven pathogenic effect, *in silico* tools predicted this change as potentially pathogenic. Unfortunately, the segregation of this variant in the family of patient 8 cannot be verified, but functional studies might be warranted to assess its biological implication.

The natural history of *SOD1*-ALS has been described in a retrospective study on a cohort of 175 patients from North America with genetically confirmed *SOD1* mutations (45). The mean age of disease onset was 49.7 years, with a median disease survival of 2.7 years. As expected (46), ALS patients with the p.Ala5Val variant presented lower disease duration, decreased ALS Functioning Rating Scale-Revised (ALSFRS-R), and higher forced vital capacity decline compared to patients without p.Ala5Val variant (45). A clinical and molecular description of a European cohort of 20 *SOD1*-ALS French patients has been recently reported by Bernard et al. (47). In addition, Chen et al. provided the natural history and mutational spectrum of an ALS cohort from China, where *SOD1* mutations represent the main genetic cause of ALS (33).

Our cohort presents some peculiar features, including extreme heterogeneity of clinical manifestations (ataxia with cognitive impairment and ALS) in family members harboring the same variation in *SOD1*, disease onset with extra-motor symptoms, and presence of a mutation in both heterozygous and homozygous state in the same family, and the latter was associated with an earlier age at onset and a more aggressive disease course (30).

The prevalence of *SOD1* mutation carriers is 3.3% in our cohort, consistent with the findings raised from previous studies led on European populations (2, 48). Although most cases presented a familial transmission, four out of 15 patients had a sporadic disease, representing the 0.8% of all sALS forms. Moreover, the mean age at the onset of the disease in sporadic forms in our cohort is 43 years, which is significantly lower than the mean age at the onset of sporadic disease in the general population.

*SOD1*-mutated ALS cases may have a broad clinical variability (49, 50) within the same family, with different ages of onset and disease progression among different members. Extra-motor system symptoms in families harboring *SOD1* mutations were frequently reported in a large Scandinavian series (49): several patients demonstrated signs of autonomic, bladder, cerebellar, and/or sensory involvement, suggesting that the disease is not confined to the voluntary motor system.

Early cognitive impairment is extremely rare in *SOD1*-mutation carriers, and it is mainly reported in the late stages of the disease (51). Indeed, patients with *SOD1*-ALS usually have early age at onset and lower motor neuron involvement and display a sparing of cognitive circuits at structural and functional neuroimaging (52). However, the common notion of “cognitively spared” ALS patients has been recently called into question by several pieces of evidence from case reports and case series (53).

Despite some exceptions, cognitive dysfunction in *SOD1* carriers is mainly characterized by frontal lobar involvement, encompassing the features of ALS/frontotemporal dementia (FTD).

Patient 1.2 presented with a multidomain cognitive impairment, associated with behavioral deficits (apathy and depression) and extrapyramidal motor symptoms, and with relative memory preservation. The most peculiar finding in this patient is the concomitant presentation of cognitive impairment and ataxia without overt signs of MND. Conversely, the proband (patient 1.1) had a lower motor neuron presentation without atypical signs and symptoms, pointing to variable expressivity of p.Gly13Arg *SOD1* mutation in the same family. We hypothesize that extra-motor symptoms might be at least partially explained by the presence of other genetic factors in the proband’s father which are able to modulate phenotypic expression.

Given that the expression of *SOD1* is not limited to motor neurons, it is not altogether surprising that some patients may present at onset non-motor symptoms and signs outside the corticospinal tract.

Our findings should potentially prompt an active screening for *SOD1* mutations in all individuals with a new ALS diagnosis, including patients with an apparently sporadic disease presentation, especially after the recent authorization of Tofersen as an early access program to all ALS patients with *SOD1* mutations. Indeed, the sporadic disease might be due to several factors, including reduced penetrance, missing diagnosis in previous generations, incomplete family history, or occurrence of *de novo* mutations.

Tofersen has been tested vs. placebo in a phase III randomized, double-blind, placebo-controlled trial (VALOR NCT02623699) (15) and is currently under investigation in an open-label extension (OLE) study (NCT03070119) in *SOD1*-ALS patients. Despite failing the primary endpoint (a 48-point measure of physical function at 28 weeks measured based on ALSFRS-R score) in the VALOR trial (54), robust and significant differences between patients treated with Tofersen vs. placebo were detectable in secondary endpoints and in the explorative clinical measures, as well as in the intermediate findings from the OLE study. Although not significant, the OLE trial detected a gap between early- and late-treated patients among *SOD1*-ALS fast progressors, highlighting the importance of a precocious therapeutic window in neurodegenerative disorders.

In this regard, the importance to offer genetic testing at least for the most common ALS causative genes both to sporadic patients and to ALS patients’ relatives should be pointed out. In particular, the high frequency in Caucasian ALS patients, the wide phenotypic heterogeneity associated with its mutations, the availability of a quick and relatively simple diagnostic test, and the recent therapeutic advances in the field make *SOD1*, alongside *C9ORF72,* the first candidate gene to be sought in patients with an MND and require that its mutations should be ruled out before pursuing next-generation sequencing (NGS) approaches.

## Data availability statement

The original contributions presented in the study are included in the article/Supplementary material, further inquiries can be directed to the corresponding author.

## Ethics statement

The studies involving human participants were reviewed and approved by Comitato Etico Milano Area 2. The patients/participants provided their written informed consent to participate in this study.

## Author contributions

DG: conceptualization, data curation, investigation, visualization, and writing—original draft preparation. PR: data curation, investigation, and writing—original draft preparation. MM: data curation and investigation. RB: investigation, methodology, and visualization. SA: investigation and methodology. GC, CG, NT, and VS: supervision and writing—reviewing and editing. AR: writing—reviewing and editing. DR: conceptualization, visualization, supervision, and writing—reviewing and editing. SC: data curation, supervision, and writing—reviewing and editing. All authors contributed to the article and approved the submitted version.

## Funding

The work was supported by Italian Ministry of Health Ricerca Corrente 2022 to GC and NT.

## Conflict of interest

The authors declare that the research was conducted in the absence of any commercial or financial relationships that could be construed as a potential conflict of interest.

## Publisher’s note

All claims expressed in this article are solely those of the authors and do not necessarily represent those of their affiliated organizations, or those of the publisher, the editors and the reviewers. Any product that may be evaluated in this article, or claim that may be made by its manufacturer, is not guaranteed or endorsed by the publisher.

## Footnotes

^1^ http://www.hgmd.cf.ac.uk

^2^ https://alsod.ac.uk/

^3^ http://databrowser.projectmine.com/
