# Supplementary material for: Clinical and molecular features of patients with amyotrophic lateral sclerosis and SOD1 mutations: a monocentric study
Source: Front Neurol. 2023 May 17;14:1169689. doi: 10.3389/fneur.2023.1169689 (PMC10230028; doi:10.3389/fneur.2023.1169689)
Supplement: Supplementary file 1 [file Data_Sheet_1.pdf]

Supplementary Material

Clinical and molecular features of patients with Amyotrophic Lateral Sclerosis and *SOD1* mutations

Supplementary tables

Supplementary Table 1. Primer sequences for *SOD1* resequencing.

Exon	Forward Primer (5'-3')	Reverse Complement Primer (5'-3')	Ampl. Size
1	CATTTTCGCGTACTGCAAAA	AAAGGGTCGCGGGGGGACGA	259bp
2	GCTGGAGGTTCACTGGCTAG	GGGTTTTAACGTTTAGGGGC	340bp
3	TTCTGTTCCCTTCTCACTGT	TCCCCTTTGGCACTTGTATT	498bp
4	CATCAGCCCTAATCCATCTGA	CGCGACTAACAATCAAAGTGA	242bp
5	TGTTGGGAGGAGGTAGTGATT	AGCAGAGTTGTGTTAGTTTTAG	674bp

Supplementary Table 2. Pathogenicity prediction and ACMG classification of variants in *SOD1* identified in our cohort.

Variant	Patients	Base change	REVEL[1]	GnomAD[2] Frequency	ACMG[3]
p.Gly13Arg	1.1; 1.2	c.37G>C	0.754	0	LP (PS4, PM2, PP2, PP3)
p.Glu22Gly	2	c.65A>G	0.845	0	P (PS4, PM2, PM5, PP2, PP3)
p.Gln23Arg	3	c.68A>G	0.785	0	LP (PM2, PM5, PP2, PP3)
p.Pro67Leu	4	c.200C>T	0.950	0	LP (PM2, PM5, PP2, PP3)
p.Pro67Ser	5	c.199C>T	0.957	3.98e-6	LP (PS4, PM2, PP2, PP3)
p.Asp91Ala	6; 7	c.272A>C	0.555	1.50e-3	VUS (PM5, PP2, PP3, BS1, BS2)
p.Ala96Thr	8	c.286G>A	0.776	0	P (PS4, PM2, PM3, PP2, PP3)
p.Leu107Val	9	c.319C>G	0.841	0	LP (PS4, PM2, PP2, PP3)
p.Leu118Val	10	c.352C>G	0.517	0	VUS (PM2, PP2, PP3)
p.Glu122Gly	11.1; 11.2	c.365A>G	0.928	0	LP (PS4, PM2, PP2, PP3)
p.Leu145Phe	12; 13; 15	c.435G>C	0.915	1.59e-5	P (PS1, PS4, PM2, PM5, PP2, PP3)
p.Leu145Ser	14.1; 14.2; 14.3	c.434T>C	0.960	0	P (PS4, PM2, PM5, PP2, PP3)

[1]REVEL score for the prediction of the pathogenicity (>0.500) of rare missense variants. Ioannidis NM et al. REVEL: An Ensemble Method for Predicting the Pathogenicity of Rare Missense Variants. Am J Hum Genet. 2016 Oct 6;99(4):877-885. Doi: 10.1016/j.ajhg.2016.08.016. PMID: 27666373.

[2]GnomAD v.2.1.1. Karczewski KJ et al. The mutational constraint spectrum quantified from variation in 141,456 humans. Nature. 2020 May;581(7809):434-443. Doi: 10.1038/s41586-020-2308-7. Website: https://gnomad.broadinstitute.org

[3]ACMG: American College of Medical Genetics and Genomics. P= pathogenic; LP= likely pathogenic; VUS = variant of uncertain significance. PS1 was assigned in case of the same amino acid change (but a different nucleotide change) of a previously established *SOD1* pathogenic variant. For PS4 criterion we considered the ALS cases reported in the following dedicated databases: ALSOD (https://alsod.ac.uk/output/gene.php/SOD1), LOVD (https://databases.lovd.nl/shared/variants/SOD1/unique) and available literature. PS4 was assigned for variants reported in at least 2 independent ALS cases and with a frequency <0.1% in controls. For PM2 criterion, we applied a threshold of (less than) 5 counts in gnomAD v.2.1.1 database (see note 2). PM5 criterion was assigned for a missense amino acid change occurring at the same position of another pathogenic *SOD1* missense change. PP2 was assigned in case of missense variant since missense variations are a common cause of disease in *SOD1*-related ALS. PP3 criterion was assigned for a REVEL score >0.5 (see note 1). BS1 was assigned for a variant with a frequency greater than 0.1% (see note 2). BS2 was assigned if the variant was observed in homozygous state in at least 2 individuals in the population database gnomAD (see note 2).
